# Nasolabial Cyst Presenting as a Painful Nasolabial Fold Swelling: A Case Report

**DOI:** 10.7759/cureus.88927

**Published:** 2025-07-28

**Authors:** Abhishek S Motimath, Tejraj Kale, Ojasvee Hiran, Radhika Pathak

**Affiliations:** 1 Oral and Maxillofacial Surgery, KLE Academy of Higher Education & Research, Belagavi, IND

**Keywords:** kledalst cyst, maxillofacial cyst, nasoalveolar cyst, nasolabial cyst, soft tissue cyst

## Abstract

Nasolabial cysts represent uncommon, non-odontogenic soft tissue lesions believed to arise from remnants of the nasolacrimal duct, and are a rare form of cysts in the maxillofacial region. They most frequently occur in women during their fourth and fifth decades of life and usually appear as a slow-growing, painless swelling located in the nasolabial fold. We present the case of a 31-year-old female patient with a six-month history of a painful, soft, fluctuant, 3x3 cm nasolabial fold swelling. CT suggested a cystic lesion, confirmed as a nasolabial cyst after intraoral excision via sublabial incision and histopathological examination. Nasolabial cysts are rare and can pose diagnostic challenges due to their nonspecific presentation, and should be considered in the differential diagnosis of nasolabial fold swellings. CT and MRI are vital for cyst evaluation. Surgical excision ensures favourable outcomes with minimal recurrence risk. This case highlights that early diagnosis and surgical management are crucial for excellent prognosis and also reinforces the importance of awareness and timely management to prevent complications and achieve optimal patient outcomes.

## Introduction

Nasolabial cysts are rare soft tissue non-odontogenic cysts that develop between the nasal vestibule and upper lip. The incidence of nasolabial cysts is 0.7% of all maxillofacial cysts [1]. Nasolabial cysts were first described by Zuckerkandl in 1882 and are also known as nasoalveolar cysts [2]. Rao coined the term “nasolabial,” offering a more accurate description than “nasoalveolar,” for these uncommon yet recognized cysts [3].

Nasolabial cysts present as unilateral, painless swellings that gradually obliterate the nasolabial fold, displace the ala nasi, and project into the nasal floor or buccal vestibule. Typically undiagnosed unless infected, they may form nasal-draining fistulas. These cysts are located lateral to the midline and the nasal septum base, appearing cystic, mobile, and unencased by bone during bi-digital examination. Their presentation underscores the importance of early clinical evaluation for diagnosis and surgical management [4].

Due to their location and presentation, nasolabial cysts can mimic facial cellulitis, periodontal abscess, acute maxillary sinusitis, or a nasal furuncle. This case report reviews our experience in the diagnosis and management of a nasolabial cyst in a 31-year-old female patient.

## Case presentation

A 31-year-old female patient presented with a six-month history of a slowly growing, painful right nasolabial swelling, causing discomfort during facial movements. The patient denied any history of trauma or surgery in that region.

On examination, the lesion was a soft, fluctuant, and well-demarcated swelling measuring 3x3 cm in the right nasolabial fold, causing asymmetry of the face. The overlying skin was intact, with no discoloration, but there was the presence of pain due to inflammation. On palpation, the swelling was tender, mobile, and not fixed to underlying structures. Intraoral examination revealed a bulge in the labial vestibule from teeth 11-14; the mucosa was normal as compared to its counterpart, with no pathological changes. Extraoral swelling was present, extending from the ala of the right nostril to the corner of the mouth. There were no associated systemic manifestations, lymphadenopathy, or nasal obstruction.

**Figure 1:**
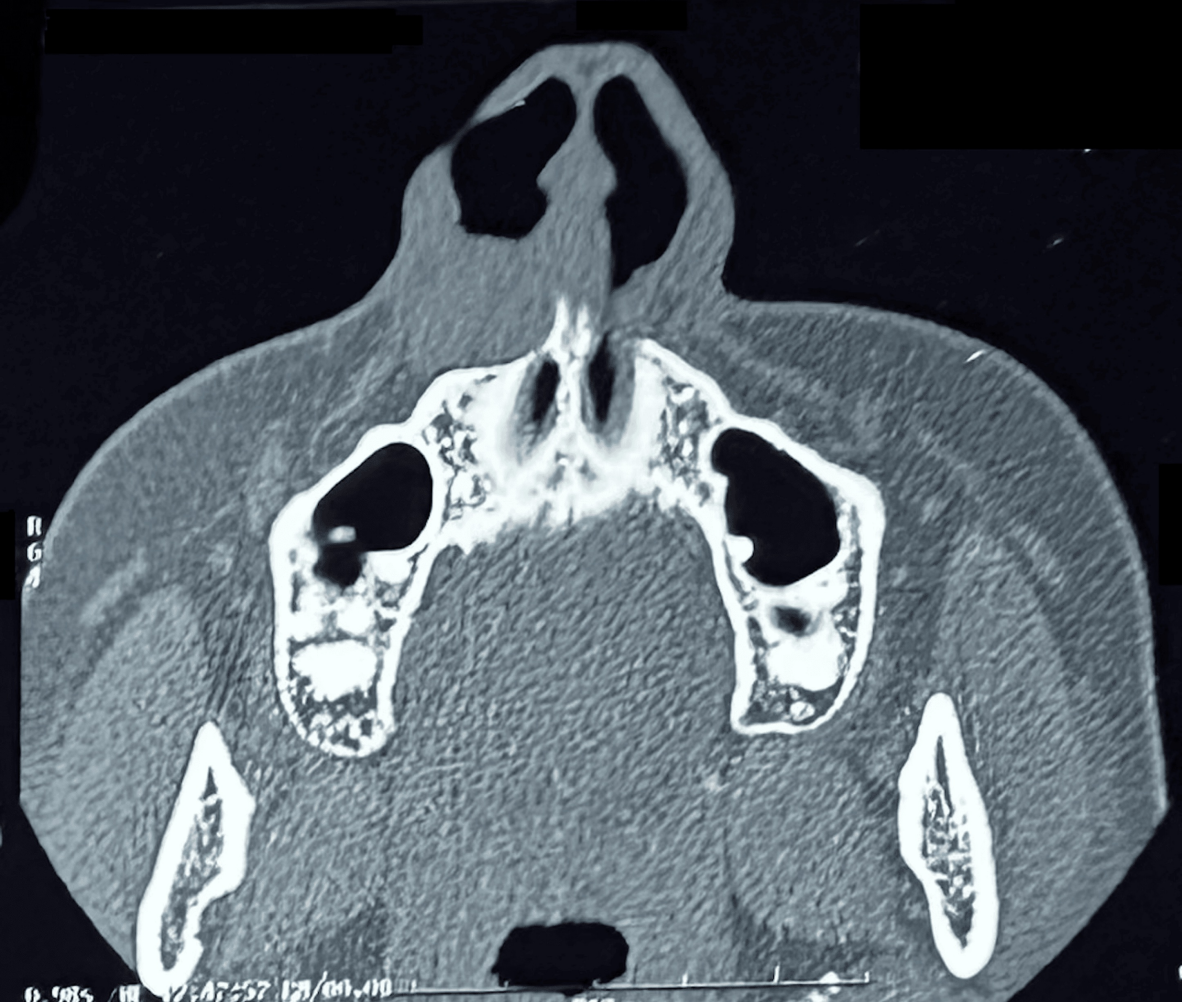
CT scan of paranasal sinuses (axial section) showing rounded mild hyper dense cystic lesion measuring ~ 2 x 1.1 cm in right nasolabial soft tissue plane. There is mild compression remodelling of underlying maxillary bone with no significant erosions

Plain CT of the paranasal sinuses was obtained, which revealed a rounded, mildly hyperdense cystic lesion measuring 2x 1.1 cm in the right nasolabial soft tissue plane. Mild compression-remodelling of the underlying maxillary bone was noted without any erosions (Figure 1). Based on the clinical and radiological findings, a provisional diagnosis of a nasolabial cyst was made.

**Figure 2:**
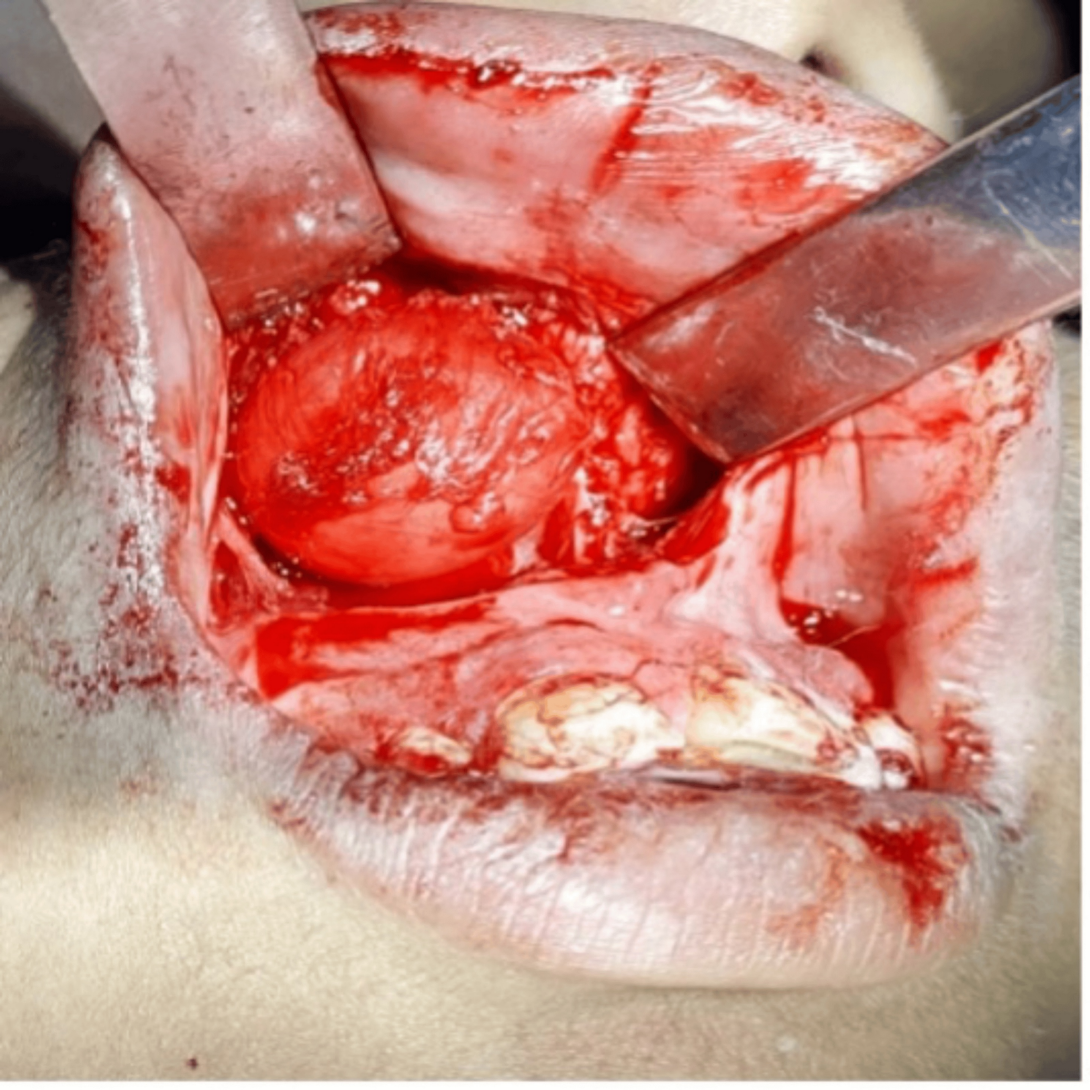
Intraoperative image reveals a round, well-circumscribed cyst with smooth, clearly defined margins, suggesting a benign nature. The cyst appears encapsulated, separated from surrounding tissues. Its distinct borders facilitate surgical excision, minimizing damage to adjacent structures and aiding in complete removal.

The patient underwent intraoral sublabial enucleation under local anaesthesia. Following aseptic protocols, a semilunar incision was made, and a full-thickness periosteal flap was reflected in the right labial vestibule, extending from the 21-14 region, 1 cm above the attached gingiva. A round, smooth, extraosseous cystic swelling superficial to the anterior maxillary wall was exposed (Figure 2), bluntly dissected from surrounding tissues, and completely excised. Hemostasis was achieved, revealing a smooth bone surface with an indentation. The incision was closed with 3-0 black braided silk, and healing was satisfactory after suture removal.

**Figure 3:**
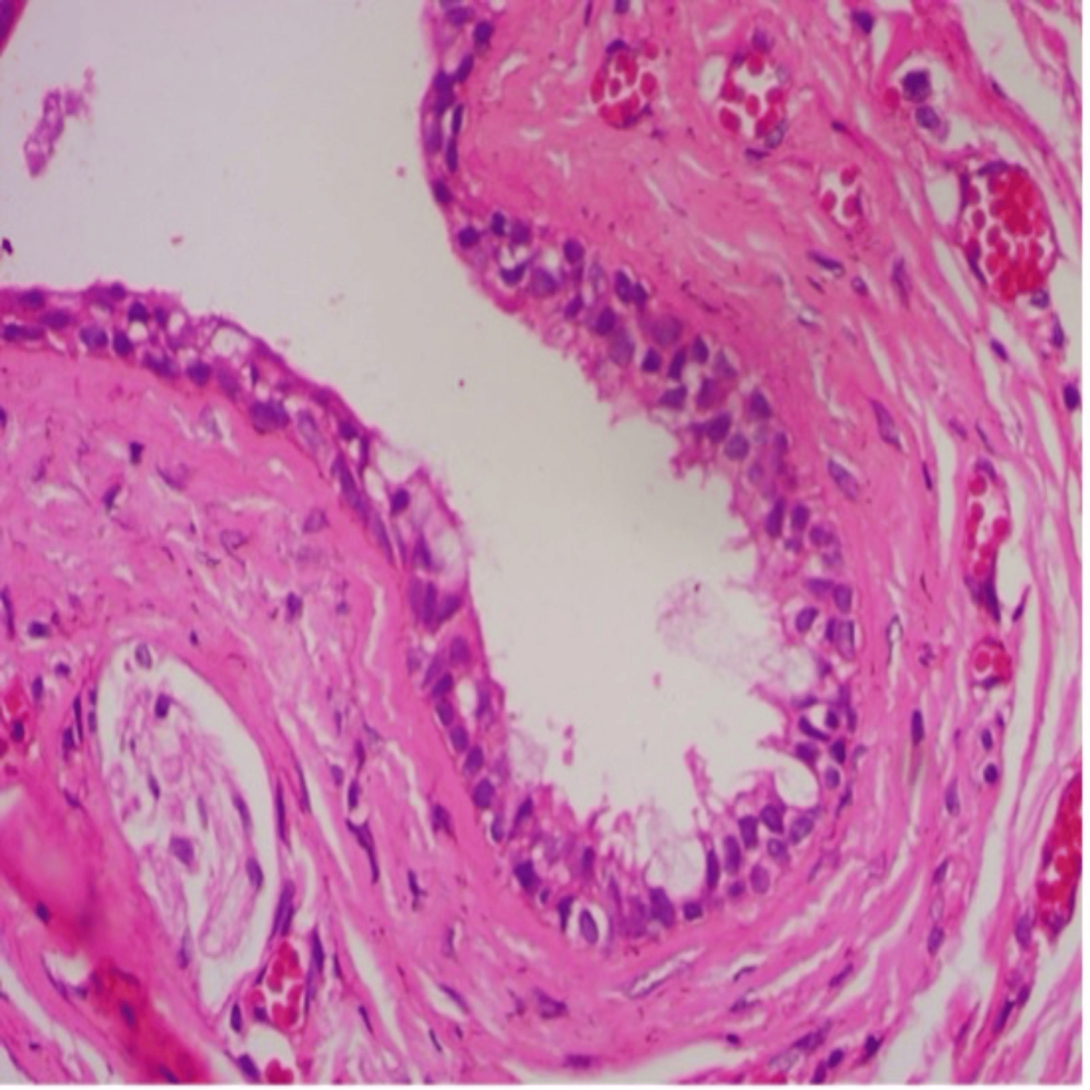
Histopathological image (H&E, 40X magnification) shows a cystic lining of pseudostratified columnar epithelium with goblet cells, supported by fibrous connective tissue . Endothelial lined blood vessels with RBCs and extravasated RBC can also be seen.

The excised sample, contained in a single piece of greyish soft tissue, underwent histopathological examination under higher magnification. It revealed cystic epithelium lined by pseudostratified ciliated columnar cells with goblet, clear, and mucous cells. Focal areas displayed epithelial transitions, mucin pooling, and papillary projections. Dense fibrous connective tissue with parallel collagen bundles indicated a cystic capsule. Scant inflammatory infiltrates, nerve and muscle sections, and numerous endothelial-lined blood vessels with RBCs and extravasation confirmed the histopathological features of a nasolabial cyst (Figure 3).

## Discussion

Nasolabial cysts are rare, comprising 0.7% of all maxillofacial cysts and 2.5% of non-odontogenic cases. Predominantly unilateral (90%), they occur bilaterally in 10% and are more common in Black women aged 40-50 years[1]. Nasolabial cysts are purely soft tissue nonodontogenic cysts, though it has been classified under jaw cysts. It is also called a mucoid cyst of the nose, nasal cyst, and sometimes even nasoalveolar cyst or Klestadt cyst [5].

The nasolabial cyst likely originates from epithelial remnants of the nasolacrimal duct, as proposed by Bruggemann in 1920 [6]. This theory explains the cyst's consistent location at the nasal floor, dismissing alternative embryological theories* *[7].

Typically, and most commonly, patients present with a painless swelling on the left side of the upper lip near the nasal alae, characterized by its very slow growth rate. These cysts range in size from 1-5 cm and rarely erode the underlying bone unless they reach a considerable size. Their submucosal location at the anterior nasal floor is both distinctive and consistent [8]. Due to an extraosseous characteristic, it expands via the gingivobuccal sulcus and expands all the soft-tissues outwards [9].

The differential diagnosis for a nasolabial cyst includes several conditions that can present with similar features, including odontogenic cysts and tumours, dermoid cyst, epidermoid cyst, mucocele, odontogenic abscess, or minor salivary gland tumours. Various imaging modalities like CT scan helps delineate the cyst’s relationship to adjacent bony structures and assess for possible bone remodeling, but cannot reliably distinguish the internal contents or cyst wall from surrounding soft tissues, limiting the differentiation from other benign soft tissue masses, whereas the multiplanar capability of MRI enables detailed evaluation of the cyst, helping distinguish nasolabial cysts from similar lesions based on characteristic signal patterns and anatomical localisation, but histopathology is must to confirm the diagnosis [4,5,7].

Ultrasonography is a convenient, office-based imaging method for diagnosing nasolabial cysts. CT, preferred for its lower cost, provides high-resolution visualization of bone and soft tissues, depicting nasolabial cysts as well-defined, low-density lesions without bone invasion. MRI, with superior soft tissue contrast, precisely evaluates cyst boundaries and contents. Increased T1 signal intensity may indicate proteins, mucus, or pus. MRI shows the cyst as a homogeneous mass with variable intensities on T1 and T2-weighted images, without contrast enhancement [10-12].

Surgical intervention for nasolabial cysts is justified in cases of pain, swelling, or nasal obstruction, particularly when these symptoms affect breathing, speech, or mastication. Persistent swelling or recurrent infections unresponsive to conservative treatment also necessitate surgery. Aesthetic concerns due to noticeable facial deformity may lead to excision at the patient’s request. Additionally, uncertain diagnoses require surgical removal for histopathological analysis to differentiate the cyst from dermoid cysts or neoplasms. 

The most frequently used method for removing nasolabial cysts is intraoral sublabial excision. This technique enables the complete removal of the cyst and can be performed under either local or general anaesthesia. Other methods for management include aspiration of cyst followed by enucleation, endoscopic-assisted modified lateral rhinotomy approach, sublabial approach with application of cryosurgery, and endoscopic transnasal marsupialization [12].

## Conclusions

Nasolabial cysts, although rare and often overlooked, should be carefully in the differential diagnosis of swellings of the nasolabial region. Accurate diagnosis is essential to differentiate these cysts from other odontogenic and non-odontogenic lesions. Advanced imaging modalities, such as CT or MRI, greatly aid in preoperative assessment and surgical planning. Complete surgical excision via an intraoral approach remains the treatment of choice, providing excellent functional and aesthetic results with minimal risk of recurrence. This case emphasizes that prompt recognition, appropriate imaging, and timely surgical intervention is critical to ensuring favorable long-term outcomes and preventing potential complications associated with delayed treatment.
